# Tumor-Derived Microvesicles Induce, Expand and Up-Regulate Biological Activities of Human Regulatory T Cells (Treg)

**DOI:** 10.1371/journal.pone.0011469

**Published:** 2010-07-22

**Authors:** Marta Szajnik, Malgorzata Czystowska, Miroslaw J. Szczepanski, Magis Mandapathil, Theresa L. Whiteside

**Affiliations:** 1 Department of Pathology, University of Pittsburgh School of Medicine, Pittsburgh, Pennsylvania, United States of America; 2 University of Pittsburgh Cancer Institute, Pittsburgh, Pennsylvania, United States of America; New York University, United States of America

## Abstract

**Background:**

Tumor-derived microvesicles (TMV) or exosomes are present in body fluids of patients with cancer and might be involved in tumor progression. The frequency and suppressor functions of peripheral blood CD4^+^CD25^high^FOXP3^+^ Treg are higher in patients with cancer than normal controls. The hypothesis is tested that TMV contribute to induction/expansion/and activation of human Treg.

**Methodology/Principal Findings:**

TMV isolated from supernatants of tumor cells but not normal cells induced the generation and enhanced expansion of human Treg. TMV also mediated conversion of CD4^+^CD25^neg^ T cells into CD4^+^CD25^high^FOXP3^+^ Treg. Upon co-incubation with TMV, Treg showed an increased FasL, IL-10, TGF-β1, CTLA-4, granzyme B and perforin expression (p<0.05) and mediated stronger suppression of responder cell (RC) proliferation (p<0.01). Purified Treg were resistant to TMV-mediated apoptosis relative to other T cells. TMV also increased phospho-SMAD2/3 and phospho-STAT3 expression in Treg. Neutralizing Abs specific for TGF-β1 and/or IL-10 significantly inhibited TMV ability to expand Treg.

**Conclusions/Significance:**

This study suggests that TMV have immunoregulatory properties. They induce Treg, promote Treg expansion, up-regulate Treg suppressor function and enhance Treg resistance to apoptosis. Interactions of TMV with Treg represent a newly-defined mechanism that might be involved in regulating peripheral tolerance by tumors and in supporting immune evasion of human cancers.

## Introduction

Tumors have the capacity to avoid immune recognition, to induce immune cell dysfunction and to escape from immune surveillance by mechanisms that are numerous and varied [1]. For example, elevated proportions of CD4^+^CD25^high^FOXP3^+^ Treg in PBMC of cancer patients have been reported, and accumulations of Treg in the tumor microenvironment are associated with reduced patient survival [1]–[4]. Recently, we have observed that membranous vesicles (MV) or exosomes released from tumor cells also referred to as TEX are biologically active, exerting potent down-modulatory effects on human T cells [5].

Exosomes or MV (30–100 nm in diameter) originate from the endosomal compartment of normal or pathological cell types when multivesicular bodies fuse with the plasma membrane [5]–[7]. Most, if not all, cells release MV. The molecular profile of MV found in body fluids resembles that seen on the surface membrane of cells from which MV originate. MV might contain mRNA or micro RNA and, therefore, could deliver genetic information to recipient cells [8], [9]. MV are involved in various cellular activities, including angiogenesis, thrombosis, coagulation, inflammation and immunity [6], [10]. MV derived from platelets exert pleiotropic stimulatory effects, activating hematopoetic and endothelial cells [11]. MV released from dendritic cells (DC) carry MHC class I and II molecules and costimulatory proteins necessary for T-cell activation [5], [6], [12]. In contrast, MV derived from tumors (TMV) inhibit functions of immune cells, facilitating tumor progression and metastasis [13], [14]. Like TMV, those derived from placenta suppress cytotoxic activity of T cells [15], [16]. Tumor-promoting activities of TMV are well documented: TMV derived from ovarian carcinomas (OvCa) sustain angiogenesis [17]; glioblastoma TMV stimulate glioma cell proliferation [6]; TMV released from tumor-activated fibroblasts promote invasion of highly metastatic prostate carcinoma cells [18]; TMV isolated from sera of patients with head and neck cancer induce apoptosis in activated CD8^+^ T cells [19], [20]; and TMV produced by prostate cancer impair NK-cell activity through down-modulation of NKG2D expression [21]. By down-regulating functions of immune cells, TMV promote tumor progression [21], [22].

We report that TMV can stimulate expansion of human CD4^+^CD25^high^FOXP3^+^ Treg [5]. This subset of immune cells is responsible for suppressing functions of conventional CD4^+^CD25^neg^ and CD8^+^ T cells [23]–[25]. Here, we examine the effects of TMV on peripheral blood CD4^+^CD25^high^FOXP3^+^ T cells obtained from healthy donors. It appears that TMV not only induce Treg, but contribute to Treg expansion and increase their suppressive functions via mechanisms involving IL-10 and TGF-β1. Our data support the existence of intercellular cross-talk between the tumor and immune cells that might regulate anti-tumor immune responses.

## Results

### CD4^+^CD25^high^FOXP3^+^ T cells in cancer patients

**Figure 1:**
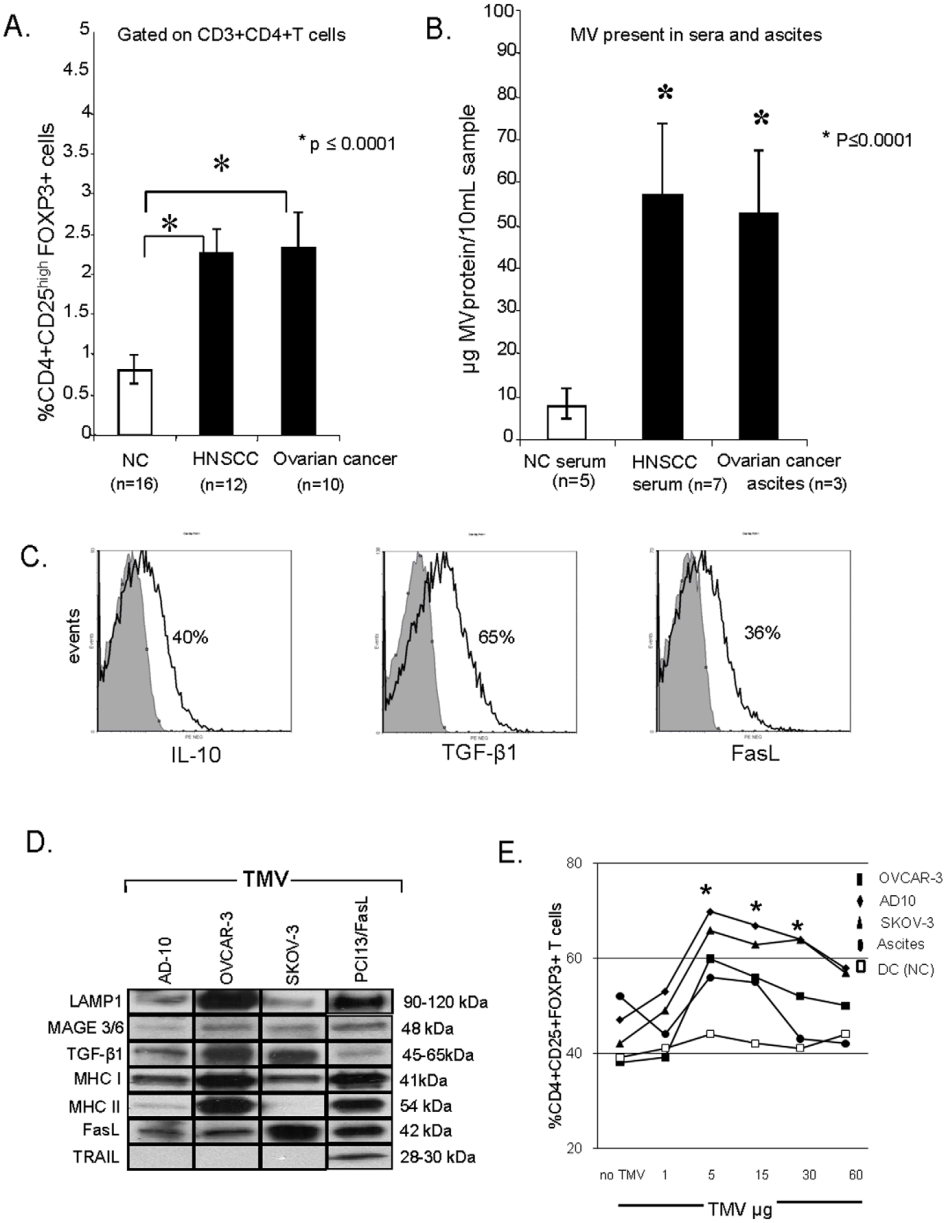
CD4^+^CD25^high^FOXP3^+^ T cells and microvesicles (MV) in cancer patients and normal controls (NC). (**A**) Percentages of CD4^+^CD25^high^FOXP3^+^ Treg in PBMC of cancer patients and NC. (**B**) the protein content/10 mL of serum or ascites in cancer patients and NC. The data in **A** and **B** are mean values ± SD. (**C**) Flow analyses of IL-10, TGF-β1 and FasL expression in MV purified from the ascites of OvCa patients and coated on latex beads. (**D**) Western blots of TMV isolated from OvCa SN. Molecular weights of the detected proteins are indicated. (**E**) Percentages of CD4^+^CD25^+^FOXP3^+^ cells in 8-day co-cultures of CD4^+^CD25^neg^ T cells with TMV obtained from various sources and used at increasing concentrations. The asterisks indicate a significant increase at p<0.05.

The frequency of CD4^+^CD25^high^FOXP3^+^ T cells was determined in PBMC obtained from HNSCC or mononuclear cells from ascites of OvCa patients by flow cytometry. The percentages of Treg were increased (p≤0.0001) in cancer patients relative to those in NC (Figure 1A).

### MV in sera and ascites of cancer patients

The protein content of MV isolated from cancer patients' sera or ascites was greater (p≤0.0001) than that of MV isolated from sera of NC (Figure 1B).

### Characteristics of TMV isolated from ascites or supernatants of OvCa cell lines

MV isolated from ascites of OvCa patients were positive for IL-10, TGF-β1 and FasL as detected by flow cytometry analyses of TMV bound to latex beads (Figure 1C). In contrast, DC-derived MV were negative for FasL and TGF-β (data not shown). The protein profiles of TMV isolated from SN of OvCa cell lines and from PCI-13/FasL SN used as a positive control [19], [20], [28] were also compared in Western blots (Figure 1D). All TMV expressed LAMP-1, confirming their endosomal origin. MV derived from ascites, OvCa cell and DC supernatants also expressed acetylcholinesterase activity (data not shown). MAGE 3/6 was detectable in all TMV as were MHC class I molecules. Expression of MHC class II molecules was low in TMV derived from SKOV-3 and AD-10, but high in OVCAR-3. A high FasL content was characteristic for all TMV, consistent with the reported expression of FasL on OvCa cells [29]. By contrast, TRAIL was not detectable. TGF-β1 was present in TMV isolated from all tested OvCa cell lines.

### TMV induce and promote proliferation of Treg

**Figure 2:**
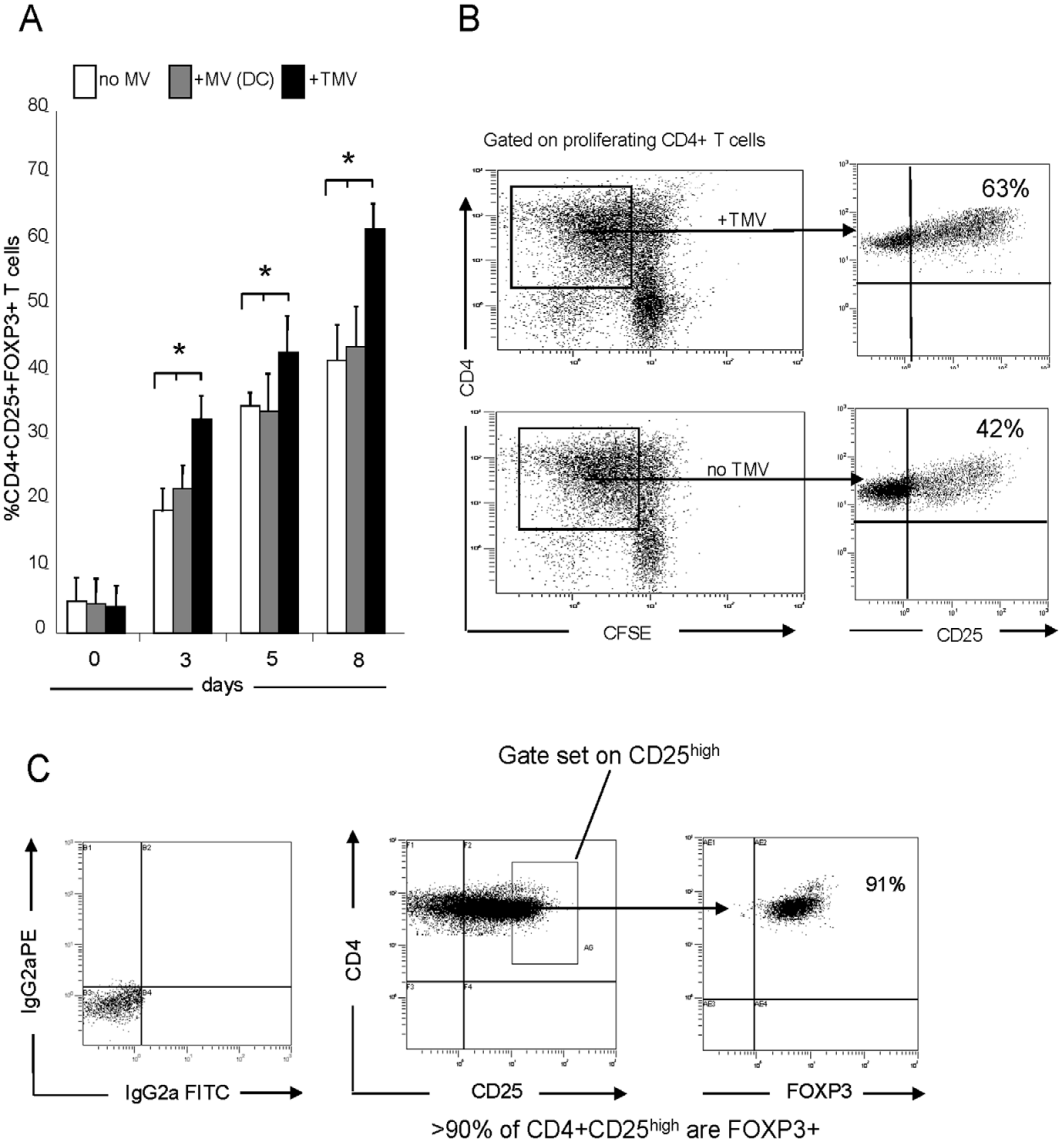
TMV promote differentiation of human Treg in culture. (**A**) Purified CD3^+^CD4^+^ T cells were labeled with CFSE and cultured as described in Materials and Methods ± TMV or DC-derived MV (5 µg/mL). On days 3, 5 and 8, the frequency of CD4^+^CD25^+^FOXP3^+^ Treg among proliferating T cells was determined by flow cytometry. The data (means ± SD) represent three independent experiments (*p<0.01). (**B**) Proliferating CD3^+^CD4^+^ T cells (squares) were tested for co-expression of CD25 in a representative co-culture ± TMV. A higher proportion of proliferating CD4^+^ T cells expressed CD25 in the co-culture with TMV than without TMV. (**C**) The proliferating CD4^+^CD25^+^ T cells in the co-cultures with TMV were evaluated for the frequency of FOXP3^+^ T cells upon gating on the CD4^+^CD25^high^ subset (see box). Over 90% of these cells also expressed intracellular FOXP3. Data are representative for one out of 6 cultures tested.

TMV (1–60 µg) were co-incubated with purified CD3^+^CD4^+^ T cells previously labeled with CFSE and activated with plate-bound OKT3, soluble anti-CD28 Ab and IL-2. The percent of CD4^+^CD25^+^FOXP3^+^ T cells increased upon co-incubation with TMV in a dose dependent manner, and the optimal TMV concentration for Treg induction was 5 µg/1×10^6^ cells (Figure 1E). MV derived from DC did not induce expansion of CD3^+^CD25^+^FOXP3^+^ Treg, as also previously reported [5]. Next, purified CD3^+^CD4^+^ T cells were labeled with CFSE, stimulated as described in Methods and cultured in the presence of TMV or DC-derived MV. The frequency of CD4^+^CD25^+^FOXP3^+^ T cells was measured on days 3, 5 and 8, it was increased at all time points relative to the baseline, and it was significantly greater (p<0.05) in co-cultures containing TMV (Figure 2A). Co-staining of proliferating CD4^+^ T cells for CD25 indicated that in the presence of TMV, over 60% of these cells were CD4^+^CD25^+^. In contrast, CD4^+^ T cells cultured without TMV contained fewer (p<0.05) CD4^+^CD25^+^ T cells (Figure 2B). Gating in these cultures on the CD4^+^CD25^high^ subset indicated that over 90% co-expressed FOXP3 (Figure 2C). These data suggest that TMV but not DC-derived MV promote the generation of CD4^+^CD25^+^FOXP3^+^ T cells in culture.

**Figure 3:**
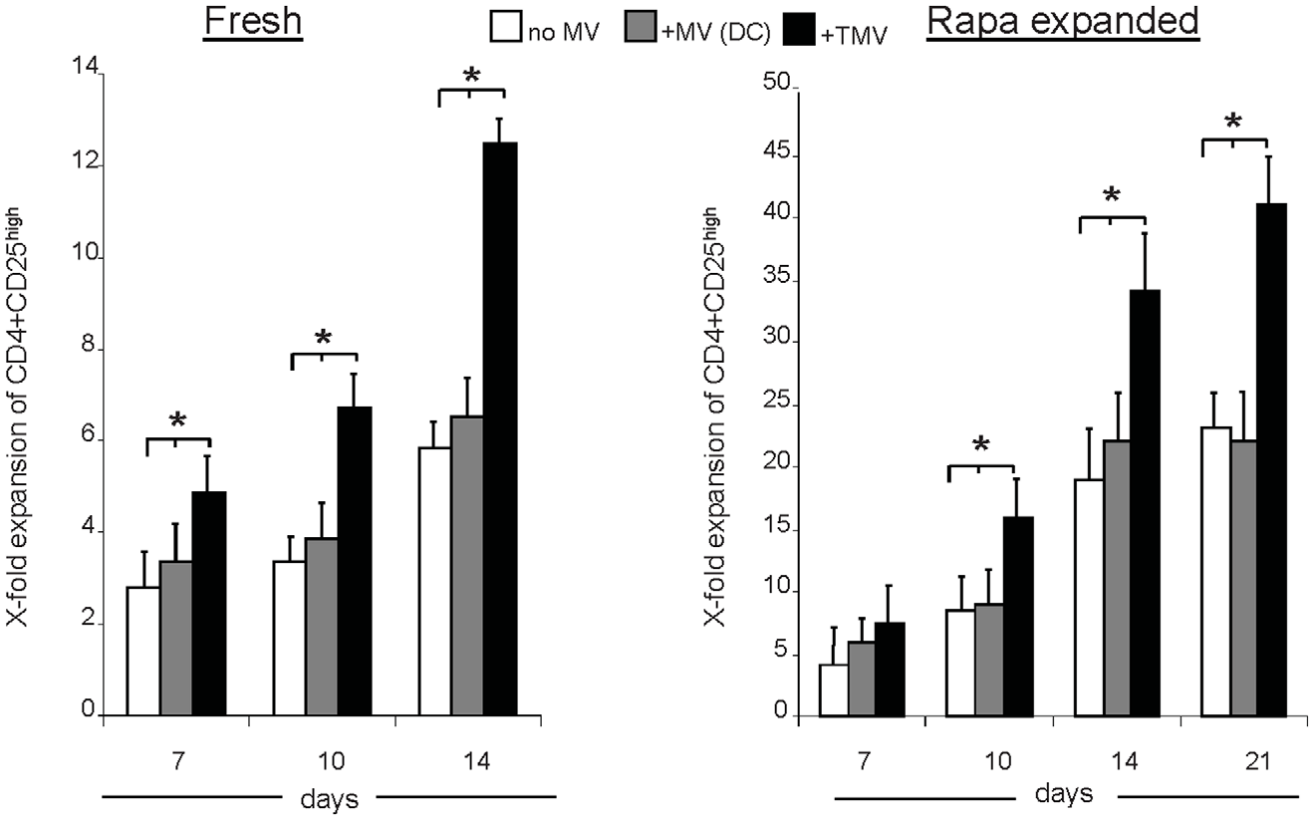
TMV promote expansion of human Treg in culture. The fold expansion of fresh (*left panel*) or rapamycin-expanded (*right panel*) CD4^+^CD25^high^ T cells to which TMV or DC-derived MV were added on day 0. Cells were stimulated with OKT3, anti-CD28 Abs and IL-2 (500 IU/mL) and cultured for 14–21 d. The data are means ± SD of six independent co-cultures. Asterisks indicate significant differences (p<0.05) between the cultures ± TMV.

To determine whether TMV helped in sustaining Treg expansion in culture, freshly isolated or rapamycin-expanded CD4^+^CD25^+^ T cells stimulated with OKT3, anti-CD28 Ab and IL-2 (500 IU/mL) were cultured ± TMV (Figure 3). The fold expansion of Treg defined as CD4^+^CD25^high^ T cells [30] was evaluated on days 7, 10, 14 and 21. In 2 week co-cultures of freshly isolated CD4^+^CD25^+^ T cells + TMV, Treg showed 12-fold mean expansion and only 3-fold mean expansion in the absence of TMV (Figure 3 *left panel*). As expected, rapamycin-expanded Treg proliferated better with the mean fold expansion of 34 on day 14 and of 40 on day 21 in TMV-containing cultures compared to 25-fold expansion at best for the cultures without TMV (Figure 3 *right panel*). The data are consistent with the conclusion that TMV promote expansion of Treg in culture.

### TMV convert CD4^+^CD25^neg^ T cells into CD4^+^CD25^+^ Treg

**Figure 4:**
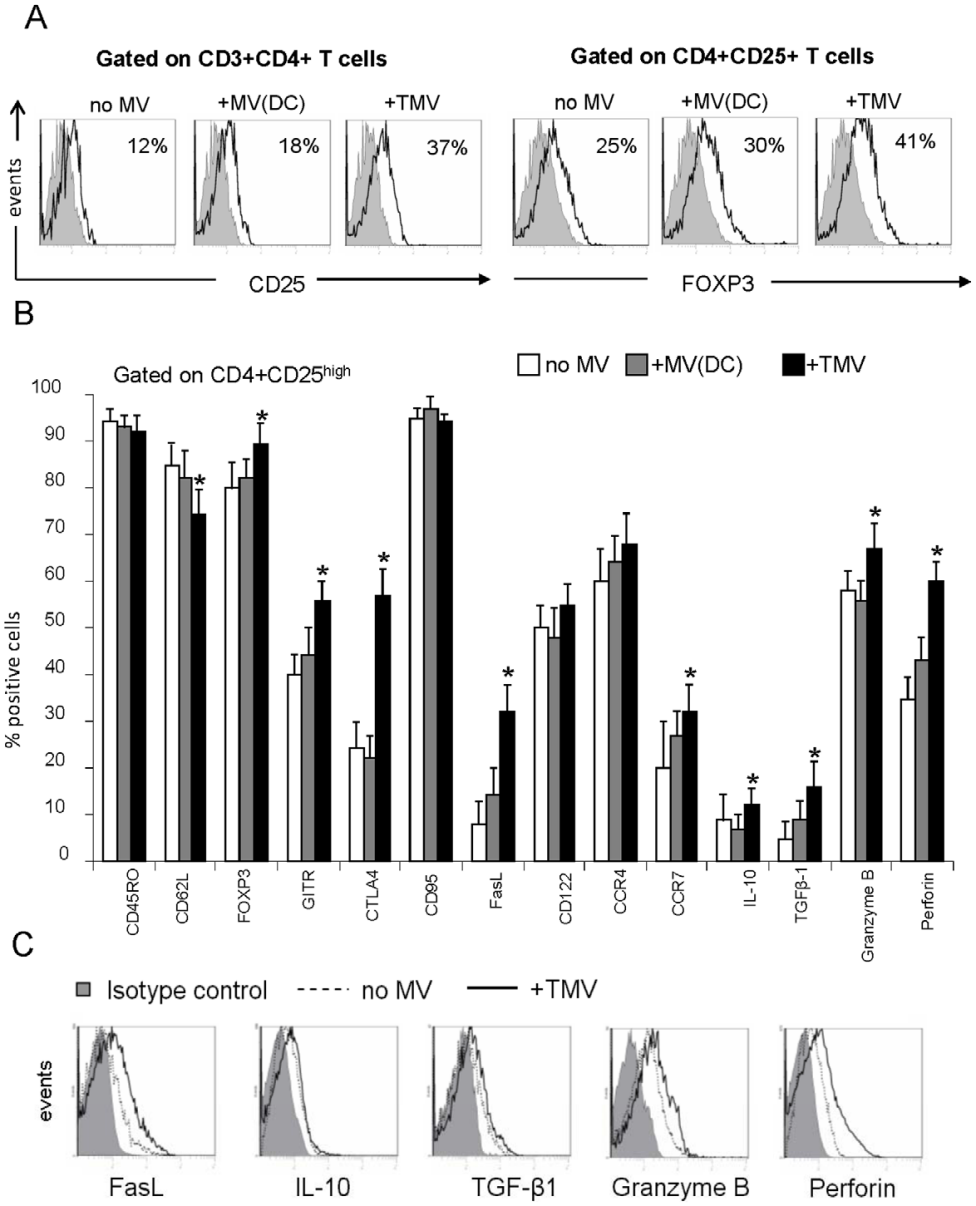
TMV Convert CD25^neg^ T cells to Treg. (**A**) Flow cytometry histograms of cultured (d5) CD4^+^CD25^neg^ T cells showing conversion of CD25^neg^ T cells into CD25^+^ T cells ± TMV or DC-derived MV (*left panel*) and expression of FOXP3 in the converted CD4^+^CD25^+^ T cells (*right panel*) in the same cultures. (**B**) A phenotypic profile of CD4^+^CD25^high^ T cells present in 7 day cultures of CD4^+^CD25^+^ T cells ± TMV or DC-derived MV. T cells were stained with various mAbs and evaluated by multiparameter flow cytometry. The gate is set on CD4^+^CD25^high^ T cells. The data are mean percentages ± SD of positive cells from three independent experiments. (**C**) MFI for FasL, IL-10, TGF-β1, granzyme B and perforin expression in CD4^+^CD25^high^ T cells cultured as described in (**B**) ± TMV. The data are representative of three independent experiments.

Freshly-isolated CD4^+^CD25^neg^ T cells were cultured ± TMV for 5 d. The percentages of CD4^+^CD25^+^ T cells were higher (p<0.05) in the presence than in the absence of TMV (Figure 4A *left panel*). After 8 days of culture, the Treg frequency further increased, suggesting that TMV promoted conversion of CD4^+^CD25^neg^ to CD4^+^CD25^+^ T cells (data not shown). The frequency of FOXP3^+^ cells was also higher in the CD4^+^CD25^+^ T cell subset cultured with TMV compared with the same cells cultured in the absence of TMV (41% vs. 25%; p<0.05) or in the presence of DC-derived MV (41% vs. 30%; p<0.05) (Figure 4A *right panel*).

### Phenotypic profile of CD4^+^CD25^+^ T cells cultured with TMV

Flow cytometry was performed on day 7 of culture to compare the phenotype of CD4^+^CD25^+^ T cells cultured ± TMV or ± DC-derived MV. By gating on CD4^+^CD25^high^ T cells, we determined the percentage of Treg and their molecular profile. In cultures containing TMV, Treg expressing GITR, CTLA-4, FasL, CCR7, TGF-β1, Granzyme B, and perforin were increased (p<0.05 for all). Fewer Treg expressing CD62L (p<0.05) compared to the cells cultured without TMV or those cultured with DC-derived MV were present (Figure 4B). The MFI for FasL, IL-10, TGF-β1, Granzyme B and perforin was also increased in Treg generated in co-cultures with TMV (Figure 4C). The phenotypic profile of CD4^+^CD25^+^ T cells expanding in the presence of TMV consistently showed enrichment in Treg expressing inhibitory cytokines and cytotoxins in comparison to cultures with no MV or with DC-derived MV.

### TMV up-regulate Treg suppressor functions

**Figure 5:**
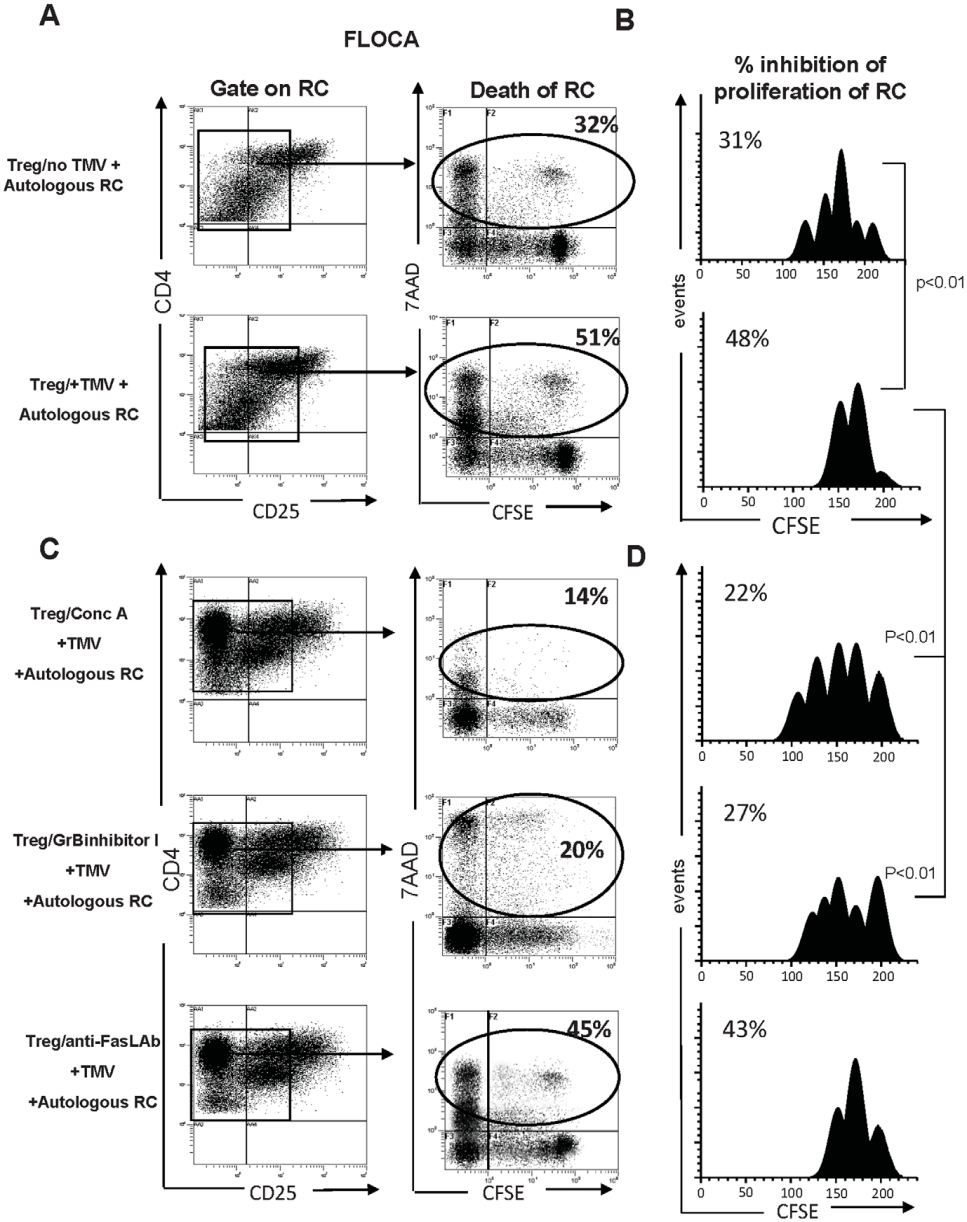
TMV increase suppressor activity of Treg. The FLOCA was used to simultaneously measure suppression proliferation of CFSE-labeled autologous CD4^+^CD25^neg^ RC and their apoptosis upon co-incubation with CFSE-negative Treg. RC cells stimulated with OKT3, anti-CD28 mAb and IL-2 (150 IU/mL) were co-cultured for 5 d with Treg pre-incubated or not with TMV. At harvest, cells were stained with 7-AAD and examined by flow cytometry. The suppressor assays were performed at the S:RC ratio of 1∶1. Treg pre-incubated with TMV induced higher levels of apoptosis (**A**) and greater inhibition of RC proliferation (**B**). The data are from one experiment of five performed. When Treg were pretreated with Concanamycin A or GrB inhibitor I and then incubated with TMV, the frequency of 7-AAD^+^ RC was lower (**C**) as was RC proliferation inhibition (**D**). Treg pretreated with FasL Ab and then incubated with TMV induced RC death (**C**) and inhibited RC proliferation (**D**).

The FLOCA was used to test whether TMV enhanced the ability of Treg to mediate suppression. This assay measures not only the inhibition of RC proliferation but also simultaneously discriminates between CFSE-labeled/7AAD^+^ (dead) and unlabeled/7AAD^neg^ (live) cells [24]. CD4^+^CD25^+^ T cells used as suppressor cells (S) were pre-incubated with TMV for 24 h and then co-cultured with autologous RC at the 1∶1 and 1∶5 ratios. The percent of dead RC was increased + TMV-treated S (Figure 5A), and concomitantly, proliferative responses of RC were inhibited (p<0.05) compared to co-cultures with untreated S (Figure 5B). We have reported that human Treg can mediate suppression using either the perforin/GrB or the Fas/FasL pathway [24], [27]. When Treg were pre-treated with Concanamycin A, which inhibits perforin activation, or with GrB inhibitor I, TMV no longer up-regulated suppressor functions of these Treg, as illustrated in Figures 5C and D. This suggests that TMV increase the ability of Treg to mediate suppression/death of RC by up-regulating activity of the perforin/GrB pathway in Treg. In contrast, anti-FasL Ab treatment of Treg had no effect on their ability to kill RC or inhibit RC proliferation ± TMV in these assays (Figures 5C and D).

### CD4^+^CD25^high^ Treg are resistant to TMV-induced death

**Figure 6:**
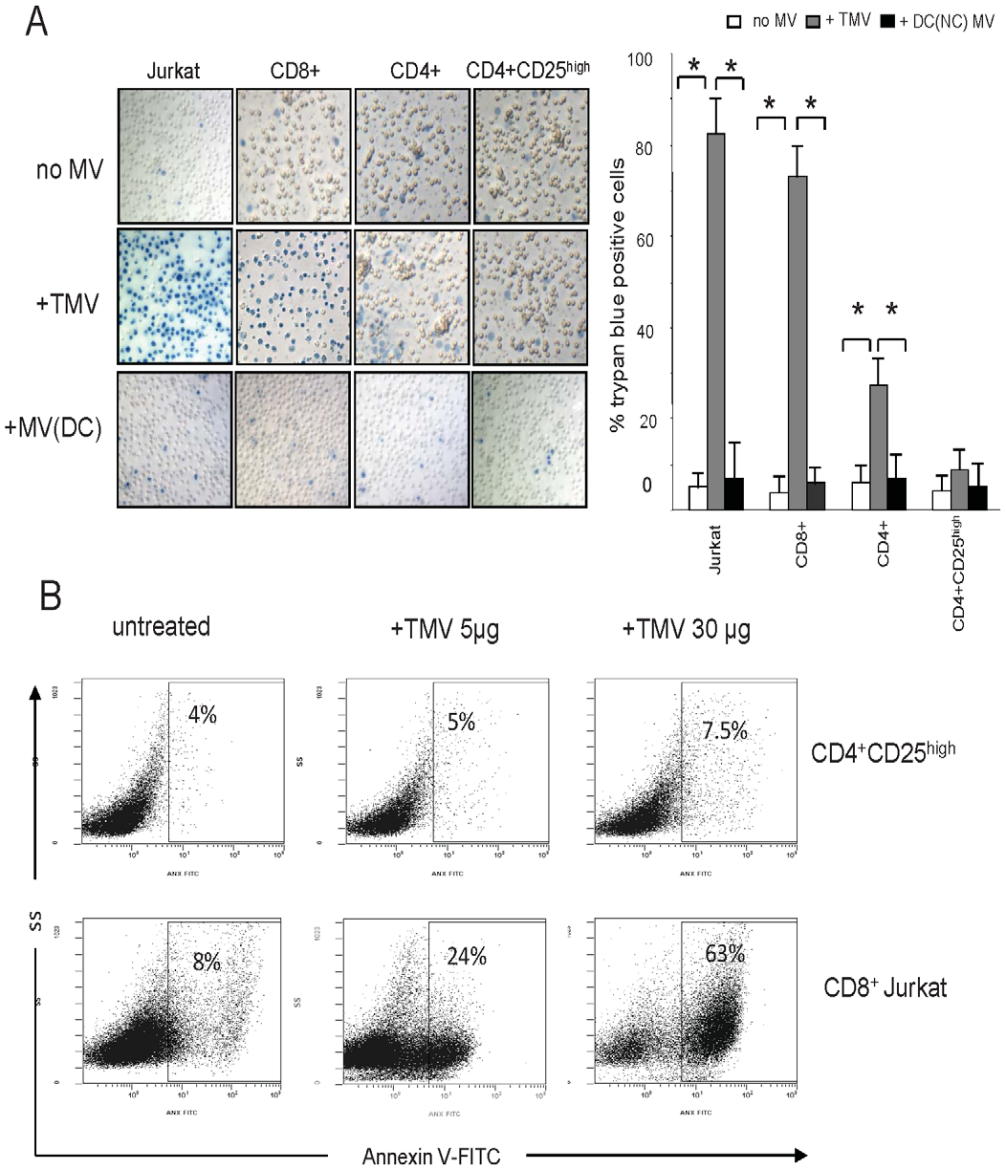
CD4^+^CD25^high^ Treg are resistant to TMV-induced death. (**A**) Trypan blue positive cells after 6 h incubation ± TMV or DC-derived MV in primary T-cell subsets and CD8^+^ Jurkat cells (mag ×200) *p<0.001. (**B**) Percentages of ANXV binding to fresh CD4^+^CD25^high^ T cells or CD8^+^ Jurkat cells incubated ± TMV for 6 h. The data are representative dot plots from one of five independent experiments.

The ability of FasL^+^ TMV to induce apoptosis of CD4^+^CD25^high^FOXP3^+^ Treg, which express both Fas and FasL [25], [30], was tested by evaluating ANXV binding to fresh or rapamycin-expanded CD4^+^CD25^high^FOXP3^+^ T cells. TMV caused apoptosis of CD8^+^ cells or control cells (Jurkat) as measured by trypan blue staining (Figure 6A) or ANXV binding to T cells (Figure 6B). In contrast, CD4^+^ T cells showed significantly lower sensitivity to TMV-induced apoptosis. Importantly, either fresh or rapamycin-expanded CD4^+^CD25^high^FOXP3^+^ T cells were completely resistant to TMV-induced apoptosis even when TMV were used at higher doses (>30 µg).

### Levels of cytokines in supernatants of Treg cultured ± TMV

SN of activated CD4^+^CD25^+^ T cells cultured ± TMV (5 µg) for 72 h were analyzed for levels of cytokines using Luminex. The co-incubation with TMV induced an increased (p<0.05) secretion of IL-1 RA, TNF-α and of inhibitory cytokines, TGF-β1 and IL-10, from Treg. In contrast, levels of IL-1α and IL-1β were not increased (data not shown).

### Treg induction is mediated by TMV-associated TGF-β1 and IL-10

**Figure 7:**
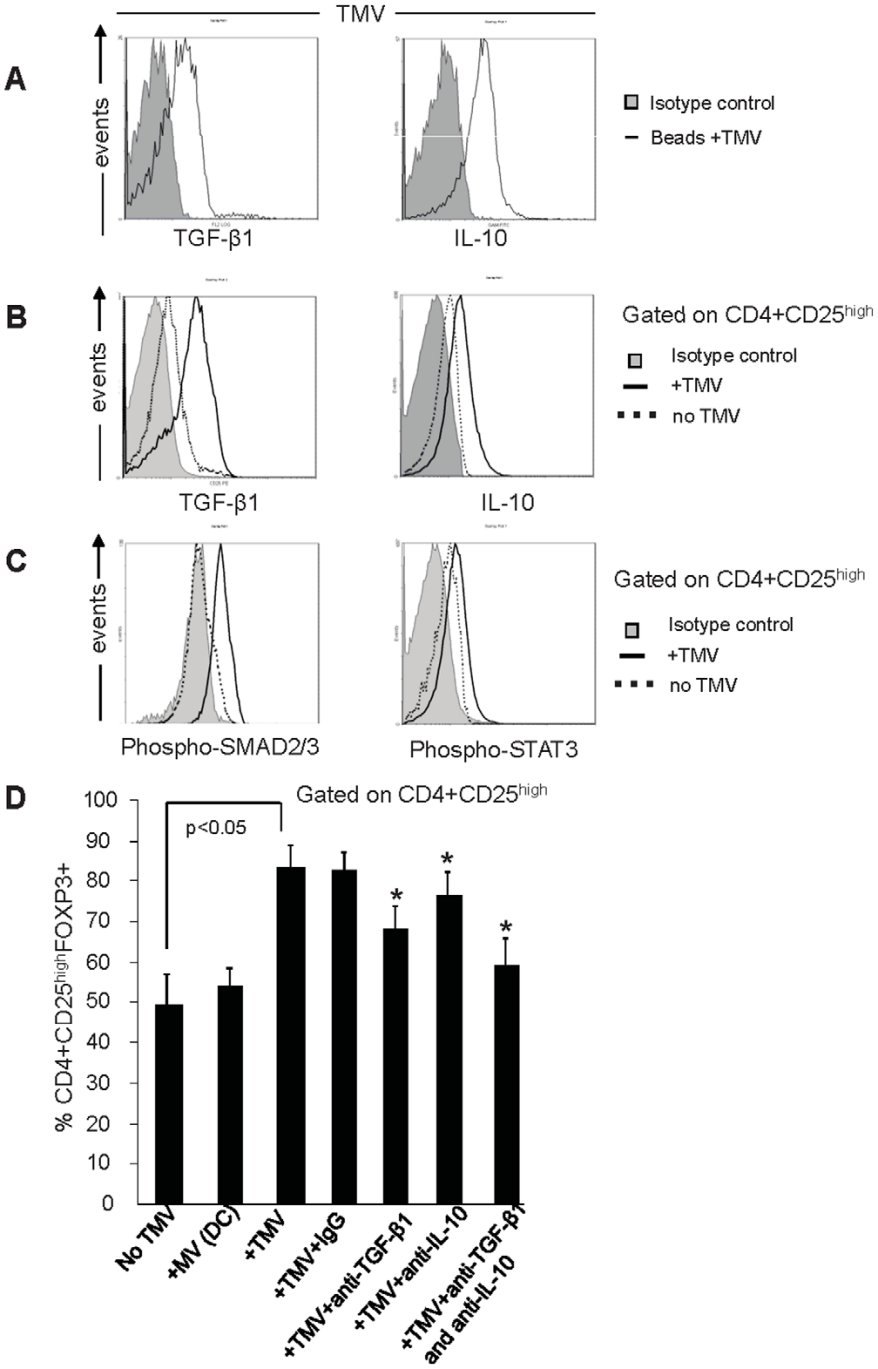
TMV-associated TGF-β1 and IL-10 promote Treg expansion. (**A**) Flow cytometry analysis of TGF-β1 and IL-10 expression on TMV purified from OVCAR-3 SN and coated onto latex beads. (**B**) CD4^+^CD25^high^FOXP3^+^ T cells were cultured with OKT3, anti-CD28 and IL-2 (150 IU/mL) +/− TMV for 72 h at 37°C in the presence of Golgistop and then stained for CD4, CD3, CD25 and intracellular TGF-β1 and IL-10. Expression of both cytokines was up-regulated in the presence of TMV (p<0.05). (**C**) SMAD2/3 and STAT3 phosphorylation in Treg before and after exposure to TMV. Representative results are from one of three independent experiments for **A**, **B** and **C**. (**D**) The percentage of CD4^+^CD25^high^FOXP3^+^ T cells increased among CD4^+^CD25^+^ T cells cultured in the presence of TMV but not DC-derived MV. Neutralizing anti-TGF-β1 and/or anti-IL-10 Abs inhibited the induction of Treg by TMV. Non-blocking IgG isotype control Abs were used as controls. Asterisks indicate decreases (p<0.05) in Treg percentages in the presence of neutralizing Abs. Results are means ± SD of three independent experiments.

Flow cytometry analyses of TMV bound to latex beads showed that TMV are positive for TGF-β1 and IL-10 (Figure 7A). When CD4^+^CD25^high^FOXP3^+^ Treg were co-incubated with TMV, expression of TGF-β1 and IL-10 was upregulated in these cells relative to Treg incubated alone (Figure 7B; p<0.05). The percentages of TGF-β1^+^ or IL-10^+^ Treg were also increased in the co-cultures with TMV (p<0.05, data not shown). In addition, intracytoplasmic expression of phosphorylated SMAD2/3 and phosphorylated STAT3 in Treg was increased in the presence of TMV relative to Treg incubated in the absence of TMV (Figure 7C). The data suggest that TMV concomitantly increase phosphorylation of the relevant transcription factors and TGF-β1 and IL-10 expression in Treg.

As reported above and illustrated in Figure 7D, in expanding cultures of CD4^+^CD25^+^ T cells, the frequency of CD4^+^CD25^high^FOXP3^+^ Treg was increased in the presence of TMV but not of DC-derived MV (p<0.05). The pre-incubation of TMV with neutralizing anti-TGF-β (20 ng/mL) or anti-IL-10 (1 µg/mL) Abs resulted in a significant reduction (p<0.5) in the percentages of CD4^+^CD25^high^FOXP3^+^ T cells (Figure 7D). When these neutralizing Abs were used in combination, the proportion of Treg in the co-cultures was comparable to controls without TMV (Figure 7D). These results suggest that *in vitro* induction of Treg by TMV is largely mediated by TGF-β1 and IL-10. When CD4^+^CD25^+^ Treg were incubated +/− recombinant IL-10 (20 IU/mL), the concentration previously determined to be optimal for Treg induction [31], the proportion of CD4^+^CD25^high^FOXP3^+^ T cells increased in the culture (p<0.05), but the absolute number of T cells did not, suggesting that IL-10 induced the conversion of CD4^+^CD25^+^ T cells to CD4^+^CD25^high^FOXP3^+^ T cells.

## Discussion

The ability to produce and release MV is a common feature of activated cells, including tumor cells [32]. We and others have reported that TMV have properties distinct from those of MV derived from normal tissue cells [5], [33]. Notably, TMV derived from human tumors inhibit functions of immune cells [5], [21], [22]. TMV present in patients' sera or malignant effusions have been associated with immunosuppressive effects mediated by these body fluids [14], [26], [34], [35]. However, the tumor origin of MV obtained form body fluids of patients with cancer was uncertain in previous studies. Here, we used SN of cultured tumor cells as a source of TMV in order to study their effects on Treg.

Proteins present in TMV define their cellular origin and biologic functions [36]. Tumor-associated antigens, e.g., MAGE 3/6, can be used as markers of TMV purified from body fluids of patients [5], while the activity of enzymes such as acetylcholinesterase serves as a measure of their biologic integrity [26]. TMV carry MHC class I and II antigens, consistent with their ability to stimulate immune cells [37], but they also bear membrane-associated death ligands such as FasL or TRAIL [13]. Therefore, TMV are able to induce apoptosis of activated CD8^+^ T cells both *in vitro* and in the circulation of patients with cancer [38], [39]. By the same token, the enrichment of TMV in the MHC class II molecules could play a role in inducing CD4^+^CD25^high^FOXP3^+^ Treg generation and/or expansion.

The increased Treg frequency and suppressor functions in the tumor and the peripheral circulation of cancer patients [2], [40] have been linked to cancer progression and shorter survival in some studies [4], [41]. Our finding that TMV promote Treg induction and proliferation and enhance their suppressor activity identifies a potential mechanism responsible for TMV-driven Treg expansion in cancer. Earlier studies indicated that TGF-β1 can promote Treg differentiation and convert CD4^+^CD25^neg^ into CD4^+^CD25^+^ Treg [42], [43]. In our hands, the induction of CD4^+^CD25^high^FOXP3^+^ Treg cells from CD4^+^CD25^+^ precursors was enhanced in the presence of TMV positive for TGF-β1 and IL-10. Further, neutralization of TMV-associated TGF-β1 and/or IL-10 with cytokine-specific Abs inhibited Treg induction, suggesting that TMV can modulate Treg frequency and functions. TGF-β1 may be more critical in this respect than IL-10, which only induced conversion of CD4^+^CD25^+^ to CD4^+^CD25^high^ Treg but not their expansion.

It has been reported that exosome-like particles (ELP) derived from thymic cells promote naïve T cell conversion into FOXP3^+^ natural (n)Treg under non-pathological conditions [44]. This observation supports the thymic origin of FOXP3^+^ nTreg in the mouse and their generation in the microenvironment enriched in TGF-β [44]. A similar TGF-β-dependent mechanism is apparently utilized by human TMV to induce conversion of CD4^+^CD25^neg^ T cells to CD4^+^CD25^high^FOXP3^+^ Treg. If Treg expansion by TMV represents one of the mechanisms of tumor-induced immune suppression, it might also explain accumulations of inducible regulatory T cells (iTreg) in cancer patients [45]. Thus, similar molecular mechanisms involving the TGF-β pathway appear to be engaged in non-pathogenic differentiation of nTreg *in vivo* [44] and in tumor-induced iTreg generation.

TMV not only induce differentiation and increase expansion but also up-regulate Treg-mediated suppression, potentially contributing to tumor escape. In TMV-treated Treg, increased expression levels of phospho-STAT3 and phospho-SMAD2/3 and of IL-10 and TGF-β1 expression as well as production may be responsible for attenuating anti-tumor immune responses in cancer patients. This cytokine-mediated suppression mechanism is known to be utilized by nTreg and iTreg [30], [45]. Our studies demonstrated that TMV also up-regulated Granzyme B, perforin and death ligands expression in human Treg, thus endowing them with the exceptional ability to mediate suppression by several distinct mechanisms [24], [27], [30]. In aggregate, our data suggest that TMV have immunoregulatory properties, and that TMV-Treg interactions represent a newly-defined escape mechanism in cancer. The TMV molecular profile, which mimics that of the membrane in the tumor from which TMV originate [5], [46], is a determining factor in their ability to mediate suppression. By specifying this profile, the tumor can subvert functions of immune cells expressing receptors for the ligands carried by TMV [5], [19]. Given the ubiquitous presence of TMV in body fluids of cancer patients and the key role Treg play in anti-tumor responses, this might represent one of the most effective mechanisms of tumor escape from the host immune system.

## Materials and Methods

### Ethics Statement

All blood samples were obtained in compliance with the University of Pittsburgh Institutional Review Board (IRB) approved research study #980633 entitled, “Peripheral blood collection from normal donors for use in immunologic assays and research studies performed in the University of Pittsburgh Cancer Institute Immunologic Monitoring and Cellular Products Laboratory.” All subjects have signed the informed consent form approved under this University of Pittsburgh (IRB) approved study (#980633).

### Cells and cell lines

The human OvCa cell lines (OVCAR3, SKOV3 and AD10) were provided by Dr S. Khlief, NIH, Bethesda, MA and were cultured in RPMI 1640 medium supplemented with 10% FCS, 2 mM L-glutamine, 100 IU/mL penicillin and 100 µg/mL streptomycin at 37°C/5% CO_2_. PCI-13, the human head and neck squamous cell carcinoma (HNSCC) cell line was retrovirally transfected with the human FasL gene as previously described [19]. Jurkat cells obtained from ATCC (Manassas, VA) were stably transfected with the gene encoding the CD8 receptor (courtesy of Dr. H, Rabinowich, University of Pittsburgh) and were cultured as previously described [20]. All cell lines were tested and found to be negative for *Mycoplasma*. Tumor cell supernatants (SN) were collected and used for TMV isolation [5]. Peripheral blood samples were obtained from untreated HNSCC (n = 12) or OvCa patients (n = 10) and healthy volunteers (NC; n = 16). PBMC were isolated by centrifugation over Ficoll-Hypaque gradients, washed in RPMI 1640 medium, counted in a trypan blue dye and immediately used for experiments.

### Antibodies for flow cytometry

The monoclonal antibodies (mAbs) used were specific for: CD3, CD4, CD25, CD62L, CD45RO, CD95, CD152(CTLA-4) (Beckman Coulter); GITR (clone FAB 689F), CCR7, CCR4 and TGF-β1 (R&D Systems. Inc.); FOXP3 (clone PCH101), perforin (Biolegend); granzyme B (clone GB111) (PeliCluster Inc.); phospho-SMAD2/3 (Cell Signaling); phospho-STAT3 (pY705) (BD Biosciences); donkey anti-rabbit IgG (Santa Cruz Biotechnology); IL-10, FasL (NOK-1.42 kDa), and isotype controls IgG1, IgG2a and IgG2b (BD Pharmingen).

### Surface and intracellular staining

Cells were stained as previously described [25]. To establish optimal staining dilutions, all mAbs were titrated using normal resting or activated PBMC. For intracellular staining, cells were permeabilized using PBS containing 0.5% (wt/v) BSA and 0.2% (v/v) saponin (Sigma Aldrich), stained with the mAbs of desired specificity or isotype control Abs for 30 min at RT, washed in buffer and analyzed by flow cytometry.

### Flow cytometry

A Beckman Coulter cytometer equipped with Expo32 software was used. Acquisition and analysis gates were restricted to the lymphocyte gate based on characteristic forward (FSC) and side-scatter (SSC) properties of the cells. FSC and SSC were set in a linear scale. For analysis, 1×10^5^ lymphocytes were acquired. Analysis gates were restricted to the CD3^+^CD4^+^, CD3^+^CD8^+^, CD4^+^CD25^high^ or CD4^+^CD25^neg^ T cell subsets, as appropriate.

### CD4^+^CD25^high^ and CD4^+^CD25^neg^ T cell isolation

CD4^+^CD25^high^ T cells from PBMC of NC were single-cell sorted using previously described gating strategy [23]–[25] with the threshold for CD25^high^ cells established at MFI of 120. A MoFlo high-speed cell sorter (DakoCytomation) was used for cell isolations. The CD4^+^CD25^neg^ and CD4^+^CD25^high^ cell fractions were collected and tested for expression of FOXP3 by flow cytometry and for viability by a trypan blue dye exclusion. The CD4^+^CD25^neg^ T cells were used as responder cells in suppressor assays. CD4^+^CD25^high^ cell purity was usually 86 to 92%, and 75–83% of the sorted cells expressed FOXP3. The sorted cells were immediately used for experiments.

### Culture of CD3^+^CD4^+^ or CD4^+^CD25^+^ T cells

CD4^+^CD25^+^ T cell were separated in AutoMACS (Miltenyi Biotec) by a two-step procedure and cultured with rapamycin, as previously described [25]. Briefly, non-CD4^+^ cells were labeled with a cocktail of biotin-conjugated Abs specific for CD8/CD14/CD19/CD16/CD56/CD123, and the labeled cells were depleted using anti-biotin Ab-coated beads. CD4^+^CD25^+^ T cells were isolated by positive selection from the pre-enriched CD4^+^ T cell fraction using beads coated with anti-CD25 Abs. Total CD3^+^CD4^+^ T cell fractions or isolated CD4^+^CD25^+^ cells were cultured in AIMV medium with plate-bound OKT3 (1 µg/mL; American Type Culture Collection), soluble anti-CD28 Abs (1 µg/mL) and IL-2 (150 IU/mL) at 37°C/5%CO_2_ in wells of 96-wells plates. On day 5, cells were transferred to wells of 48-well plates and restimulated with anti-CD3/anti-CD28 mAbs-coated beads and 1,000 IU/mL IL-2. Rapamycin (1 nM; Sigma-Aldrich) was added to the cultures on day 7. After three weeks of culture, cells were washed and beads were removed. Among cultured CD25^+^ T cells, 80 to 91% expressed FOXP3.

### Isolation of TMV

TMV were isolated from ascites of OvCa, blood of HNSCC patients or SN of tumor cell lines as previously described [5], [19]. Briefly, the concentrated SN were fractioned using size exclusion chromatography and ultracentrifugation. Aliquots (10 mL) of concentrated SN were applied to a Sepharose 2B (Amersham Biosciences) column. Total protein of collected fractions was monitored by absorbance at 280 nm. The exclusion peak fractions (>50 million kDa) were centrifuged at 105,000 x *g* for 2 h at 4°C. The pellet was resuspended in 300 µL of PBS. The protein concentration was estimated by the Lowry's protein assay (Bio-Rad Laboratories) with BSA used as a standard.

### Acetylcholinesterase activity in TMV

Using a previously described assay [26], 25 µL of TMV were suspended in 100 µL of PBS and incubated with 1.25 mM acetylocholine and 0.1 mM 5,5 dithiobis (2-nitrobenzoic acid) in a final volume of 1 mL. After 15 min of incubation at 37°C, changes in absorption were monitored at 412 nm.

### Co-incubation of T cells and TMV

Isolated, fresh or cultured CD4^+^CD25^high^ FOXP3^+^, CD4^+^CD25^neg^ or Jurkat T cells were incubated with varying concentrations of TMV (5 to 60 µg/mL) for different time periods at 37°C/5% CO_2_. The viability of harvested cells was determined using a trypan blue dye exclusion. The cells were phenotyped and assessed for functions as described below.

### Apoptosis assays

Annexin V binding to Treg, Jurkat cells or primary T cells co-incubated with TMV for 6 h was measured by flow cytometry. Following surface staining with mAbs for CD3, CD8, CD4 or CD25, the cells were resuspended in ANX-binding buffer and incubated with FITC-conjugated ANXV for 15 min on ice. The cells were analyzed by flow cytometry within 30 min of staining.

### Flow cytometry-based cytotoxicity assay (FLOCA)

Induction of apoptosis and suppression of responder cells (RC) proliferation mediated by CD4^+^CD25^high^ T cells before and after exposure to TMV was analyzed using the FLOCA [24]. CFSE-labeled autologous RC were cultured with Treg at various Treg/RC ratios in the presence of soluble OKT-3 (1 ug/mL), and anti-CD28 (1 µg/mL) mAbs and IL-2 (150 IU/mL) for 5 d. The harvested cells were stained with anti-CD25 and anti-CD4 Abs and incubated in PBS containing 20 µg/mL of 7-amino-actinomycin D (7-AAD; Calbiochem) for 20 min at 4°C in the dark and immediately analyzed by flow cytometry.

All CFSE data were analyzed using the ModFit software provided by Verity Software Hause. The percentage of suppression was calculated based on proliferation index (PI) of RC alone compared with the PI of cultures containing RC and Treg. The program determines the percent of cells within each peak and the sum of all peaks in the control culture is taken as 100% of proliferation and 0% of suppression.

### Analysis of Treg-mediated suppression of RC proliferation ± TMV

The FLOCA was performed under various conditions to determine the potential involvement of the granzyme/perforin or Fas/FasL pathways in TMV-mediated suppression of CD4^+^ RC proliferation. Treg were pre-treated as indicated below before co-culture with RC:

- Concanamycin A (Sigma Aldrich) was used at the concentration of 100 nM for 2 h at 37°C to block perforin activation within the lytic granules.
- GrB inhibitor I, Z-AAD-CMK (Calbiochem) was used at the concentration of 250 µM/mL for 2 h at 37°C to neutralize GrB activity.
- Anti-human FasL-neutralizing Ab (NOK-1; BioLegend) or isotype control Abs were used at 0.5 µL/100 µL for 2 h at 37°C to block FasL expression [24].

The optimal concentrations of the inhibitors were pre-determined as previously described [27]. Control cells were incubated with medium alone. The ability of the pre-treated Treg to induce RC death or suppress their proliferation was measured by FLOCA after 5 d of co-incubation +/− TMV.

### Western blots

TMV were analyzed by Western blots as previously described [20] following lysis in ice-cold lysis buffer containing a protease inhibitor cocktail (Pierce Chemical). TMV homogenates were boiled for 5 min in 5× Laemmli buffer, and proteins were separated by SDS-PAGE. Abs to LAMP-1 (Cell Signaling), MAGE 3/6 (provided by Dr. Spagnoli, Basel, Switzerland), TGF-β1 (Cell Signaling), MHC class I (clone: HC-10) and class II (LGIII 612.14) (provided by Dr. Soldano Ferrone, Pittsburgh, PA), FasL Ab-3 (Oncogene) and TRAIL (Cell Signaling) were used. Blots were evaluated with a SuperSignal detection system (Pierce Chemical).

### Flow cytometry analysis of TMV

TMV preparations (5–10 µg) were incubated with 5 µL of aldehyde/sulfate latex beads (4 µm, Inerfacial Dynamics) for 20 min at 20°C. TMV-coated beads (20 µL) were incubated with anti-TGF-β1-PE (R&D Systems, Inc.) or unconjugated anti-IL-10 (Abcam) Ab for 30 min at 4°C plus an incubation with FITC-conjugated secondary Abs (Santa Cruz) and analyzed by flow cytometry. Controls included isotype-matched Abs and fluorescence intensity was normalized for each Ab based on control values.

### Cytokine expression/production by TMV-treated Treg

Intracellular TGF-β1 and IL-10 expression by CD4^+^CD25^high^ T cells co-incubated or not with TMV was tested. Cells cultured with OKT3, anti-CD28 Ab and IL-2 (150 IU/mL) were incubated ± TMV for 24 h at 37°C in the presence of Golgistop (BD Pharmingen) and after staining for CD4, CD3, CD25, TGF-β1 or IL-10 were tested by flow cytometry.

The levels of IL-1α, IL-1β, IL-1RA, TNF-α, IL-10 and TGF-β were measured in SN of CD4^+^CD25^+^ T cells co-incubated ± TMV in 48-well plates at 3×10^5^ cells/well in 500 µL of medium for 72 h. SN were collected and tested by Luminex using reagents purchased from the Biosource International. The assay sensitivity varied from 5 to 15 pg/mL.

### Statistical analysis

Data were summarized by descriptive statistics (mean ± SD for continued variables and frequency or percentage for categorical variables). Statistical analyses were done using the paired and unpaired two-tailed Student's *t* tests. p<0.05 was considered to be significant.
